
JOURNAL INFORMATION
==============================
NLM Title Abbreviation: ScientificWorldJournal
Journal ID: ScientificWorldJournal
NLM Journal ID: 101131163
Journal ID: TSWJ

The Scientific World Journal

ISSN: 2356-6140
EISSN: 1537-744X
Publisher: Wiley

ARTICLE INFORMATION
==============================
PMCID: PMC6009328
PMID: 29973782
DOI: 10.1100/tsw.2002.8
Article ID: 289126
Article version: 1
Subjects: Short Report

Analysis of the Human 3’-Phosphoadenosine 5’-Phosphosulfate Synthase Genes

Campbell W.G.
Venkatachalam K.V. *
Department of Biochemistry, College of Medical Sciences, Health Profession Division, Nova Southeastern University, Ft. Lauderdale, FL 33328-2018, USA
*K.V. Venkatachalam: venk@nova.edu
Publication date: 2002
Electronic publication date: 2002 Apr 9
Volume: 2
First page: 15
Last page: 16
Copyright: Copyright © 2002 W.G. Campbell and K.V. Venkatachalam.
Copyright year: 2002
License: This is an open access article distributed under the Creative Commons Attribution License, which permits unrestricted use, distribution, and reproduction in any medium, provided the original work is properly cited.
License URL: https://creativecommons.org/licenses/by/3.0/

==============================
Advances in Gene Technology: The Genome and Beyond –
Structural Biology for Medicine (Proceedings of the 2002 Miami
*Nature Biotechnology* Winter Symposium)
*TheScientificWorld* 2002, 2(S2), 15–16
ISSN 1532-2246; DOI 10.1100/tsw.2002.8

### ANALYSIS OF THE HUMAN 3'-PHOSPHOADENOSINE 5'-PHOSPHOSULFATE SYNTHASE GENES

W.G. Campbell and K.V. Venkatachalam
Department of Biochemistry, College of Medical Sciences, Health Profession Division, Nova Southeastern University, Ft. Lauderdale, FL 33328-2018
(venk@nova.edu), Tel: (954) 262-1335

**INTRODUCTION.** PAPS is the universal sulfonate donor compound. PAPS synthase (PAPSS) catalyzes the formation PAPS in two concerted steps. In the first step $SO_4^-$ combines with ATP to form adenosine 5'-phosphosulfate (APS) and PPi catalyzed by ATP sulfurylase domain. In the second step APS combines with another molecule of ATP to form PAPS and ADP catalyzed by APS kinase domain. In higher eukaryotes APS kinase domain (aa 1-268) and ATP sulfurylase domain (aa 220-623) are fused together into a single polypeptide termed PAPS synthase (PAPSS) (1). cDNA of the two isoforms PAPSS1 (accession # U53447) and PAPSS2b (accession # AF 150754) from human have been molecularly cloned and characterized[1]. PAPS synthase genes have been localized to chromosome 4q24 (PAPSS1) and chromosome 10q22.3-10q25.1 (PAPSS2)[1,2].

**METHOD.** Using the GenBank human genome depository from the NCBI server, DNA sequence analyses were performed. Exon-intron junctions were verified using consensus donor and acceptor splice sites.

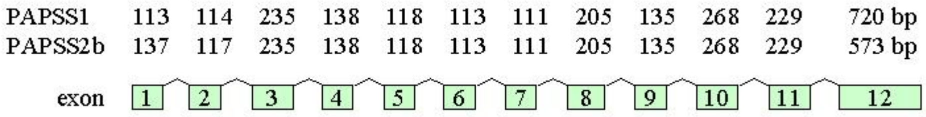

**RESULTS.** The gene sequence analysis of PAPSS1 and PAPSS2b reveals conservation in the size of the coding regions of the exons (Figure). The number of nucleotides (nt) in PAPSS1 of the exons (ex) were (ex1 = 113, ex2 = 114, ex3 = 235, ex4 = 138, ex5 = 118, ex6 = 113, ex7 = 111, ex8 = 205, ex9 = 135, ex10 = 268, ex11 = 229, ex12 = 720). From ex2 until ex11 both PAPSS1 and PAPSS2b evinced same size except there was an additional 3 nt in ex2 of PAPSS2b. The ex1 of PAPSS1 contained 54 nt of 5'-UTR and 59 nt of coding sequences, whereas PAPSS2b contained 110 nt of 5'-UTR and 27 nt of coding sequences. Ex12 of PAPSS1 contains stop codon at nt 138 and a 3'-UTR length of 582 nt. Likewise ex12 of PAPSS2b contains a stop codon at nt 194 and a 3'-UTR of 379 nt. The APS kinase-ATP sulfurylase domain requires a mandatory intervening sequence region (aa ~220-268) that is located mainly in ex6. PAPSS2b promoter contains a putative TATA box (TATACCT) at nt -18 and a GC box (GGGCGG) at –94 nt. PAPSS1 gene has a TATA-less promoter with two GC boxes at –21 and –34 nt.

**DISCUSSION.** PAPSS plays a pivotal role in producing the universal sulfonate donor compound PAPS that are required for all sulfotransferase reactions. PAPSS is a bifunctional enzyme, comprised of $NH_2$-terminal, APS kinase domain (aa 1-268) and a COOH-terminal, ATP sulfurylase domain (aa 220-623) as characterized in PAPSS1 protein[1]. The isoforms PAPSS1 and PAPSS2b are differentially expressed in various tissues[1,2]. The sizes of ex2 through ex11 of PAPSS1 and PAPSS2b are precisely placed between introns, although the chromosomal location is different. Intervening sequences (aa 220-268) located between APS kinase and ATP sulfurylase domains are coded mainly by ex6 in both genes. Cloned and expressed proteins of PAPSS1 exhibit Michaelis-Menton kinetics and PAPSS2b a sigmoidal response, with varying [ATP] *vs.* v/Vmax (unpublished). From gene analysis PAPSS2b is the inducible form and PAPSS1 the house keeping form, which is in agreement with the kinetics observed with reference to ATP. Understanding the proper structure/functions of these isoforms of PAPSS is crucial, since it plays a pivotal role in various biochemical processes. Failure to produce normal levels of PAPS can result in severe cartilage and bone defect [spondyloepimetaphyseal dysplasia (SEMD)][2] and perhaps even more severe defects, depending on the types of tissues that are affected.

**REFERENCES**

1. Venkatachalam, K.V., Akita, H., and Strott, C.A. (1998) J.Biol.Chem 273, 19311–19320.
2. ul Haque, M.F., King, L.M., Krakow, D., Cantor, R.M., Rusiniak, M.E., Swank, R.T., Superti-Furga, A., Haque, S., Abbas, H., Ahmad, W., Ahmad, M., and Cohn, D.H. (1998) Nat. Genet. 20, 157–162.
